# Changing the Diameter of the Bone Tunnel Is More Effective Than Changing the Tunnel Shape for Restoring Joint Functionality After ACL Reconstruction

**DOI:** 10.3389/fbioe.2020.00173

**Published:** 2020-12-31

**Authors:** Huizhi Wang, Min Zhang, Cheng-Kung Cheng

**Affiliations:** ^1^School of Biological Science and Medical Engineering, Beihang University, Beijing, China; ^2^Beijing Advanced Innovation Center for Biomedical Engineering, Beihang University, Beijing, China

**Keywords:** ACL reconstruction, bone tunnel, biomechanics, finite element analysis, graft diameter, knee joint

## Abstract

The clinical implications of changing the shape of the bone tunnel for Anterior cruciate ligament reconstruction (ACLR) is controversial and few studies have reported on the long-term prevalence for osteoarthritis. As such, this study aims to evaluate the effect of tunnel shape on joint biomechanics. Finite element models of an ACLR were constructed with different shapes (circular, oval, rounded rectangular, rectangular, and gourd-shaped) and diameters (7.5, 8.5, and 9.5 mm) for the bone tunnel. A combined loading of 103 N anterior tibial load, 7.5 Nm internal tibial moment and 6.9 Nm valgus tibial moment was applied at a joint flexion angle of 20°. Joint kinematics and the strain energy density (SED) on the articular cartilage were compared among the different groups. The results showed that conventional ACLR (circular tunnel) lead to an increase in joint kinematics over the intact joint, a lower ligament force and a higher SED on the lateral tibial cartilage. ACLR using the other tunnel shapes resulted in even greater joint kinematics, lower graft force and greater SED on the lateral tibial cartilage. Increasing the tunnel diameter better restored joint kinematics, graft force and articular SED, bringing these values closer to those from the intact knee. In conclusion, increasing the tunnel diameter may be more effective than changing the tunnel shape for restoring joint functionality after ACLR.

## Introduction

Anterior cruciate ligament reconstruction has long been a standard treatment for ruptured ACLs, but numerous studies have also reported on the inevitable development of knee OA at long-term follow up (Barenius et al., 2014; Lecoq et al., 2018). The primary reason for the development of OA is a failure to adequately rebuild the complex anatomy of the ACL insertion sites, including its shape and diameter. As reported by Ferretti et al. (2011), 50% (4 in 8) of subjects studied had an oval-shaped tibial insertion, while the other 50% had a rounded triangular shaped tibial insertion. For the femoral side, studies have shown that most patients have an oval-shaped insertion site (Petersen and Zantop, 2006). The area of the ACL at its insertion site has also been reported to be over 3 times larger than the cross-sectional area at its midsubstance (Harner et al., 1999). Westermann et al. (2013) reported that increasing the tunnel diameter could efficiently decrease anterior-posterior tibial translation and reduce articular stress. Alternatively, ACLR using different shapes for the tunnel aperture has been used to better restore the anatomy of the ACL insertion site. Felmet (2011) performed an ACLR using a circular femoral tunnel and an oval tibial tunnel, and reported good or excellent clinical outcomes at 6 months after surgery. Noh et al. (2011) used a gourd-shaped tibial tunnel to mimic the different areas of the tibial insertions of the anteromedial and posterolateral bundles, but found no significant difference in functionality or subjective IKDC score in comparison to a conventional ACLR at 2 years follow up. Using rectangular tunnels for ACLR, Shino et al. (2012) reported “acceptable” results in ROM, IKDC and KT-1000 at 2 years after surgery. Zhang et al. (2019) reported on the use of a rounded rectangular tibial tunnel and declared a better outcome than conventional ACLR in terms of the Tegner score, pivot shift test and graft maturity at 2 years after surgery.

As is evident from the studies above, the clinical benefit of using different shapes for the bone tunnel is controversial, and there is a lack of long-term reports on the incidence of OA. Apart from the shape of the tunnel, another factor that sets these studies apart and could have a considerable impact on the clinical outcome is variation in the procedure, such as the location of the bone tunnels, graft strength, and fixation method. However, few controlled biomechanical studies have explored the effectiveness of using different tunnel shapes for ACLR.

The aim of this study is to evaluate the effect of the shape of the bone tunnel aperture on joint biomechanics after ACLR. This study used a finite element model of a cadaveric human knee joint to evaluate the effect of tunnel shape and size on joint kinematics, graft force and the articular SED following ACLR. The strain energy is a sum of squares of the stress/strain components weighted by the anisotropic elastic components, thus has been considered as a suitable indicator for the development of OA in articular cartilage (Cowin, 1990). This study hypothesized that the diameter of the tunnel has a greater influence on joint stability than the tunnel shape, and that by increasing the tunnel diameter the ACL force and articular SED could be brought closer to those from a healthy knee. It was also hypothesized that a rounded rectangular shape for the bone tunnel aperture is the best shape for improving joint biomechanics after ACLR. The results obtained from this study may provide clinical guidance on choosing a suitable tunnel shape for improving the outcome of ACLR.

## Materials and Methods

### Finite Element Model of a Knee Joint

**FIGURE 1:**
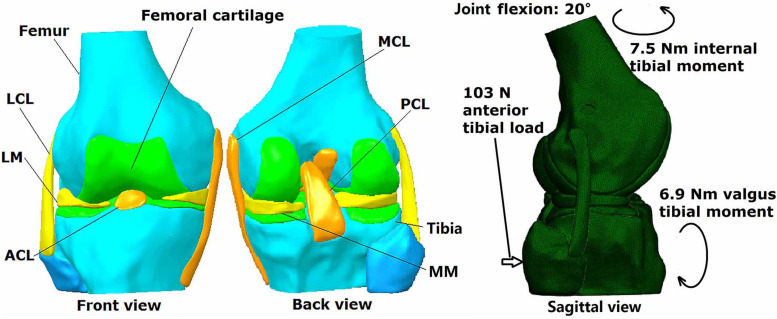
Three dimensional finite element model of human cadaveric knee joint with loading conditions.

A three dimensional finite element model of a human cadaveric knee (male, 45 years) was built and validated in a previous study using Abaqus/CAE 6.14-2 (Simulia, Inc., United States) by the authors (Wang et al., 2019), and was thus used for the current study. The model was reconstructed from MRI images (resolution: 0.2 mm; TE = 26.3 ms, TR = 53 ms) using Mimics 10.01 (Materialise NV., Leuven, Belgium) and consisted of bones, cartilage, menisci and ligaments (ACL, PCL, MCL, and LCL) (Figure 1). Four-node tetrahedron elements were used to segment the model in HyperMesh 12.0 (Altair Engineering, Tokyo, Japan) and the mesh convergence test resulted in an element size of 1 mm, with a total number of 659,251 elements for the entire model. Mechanical properties of the tissues were defined in Abaqus/CAE 6.14-2 (Simulia, Inc., United States) according to literature. Bones (Young’s modulus = 0.4 GPa, Poisson’s ratio *v* = 0.33) and cartilage (Young’ modulus = 5 MPa, Poisson’s ratio *v* = 0.46) were assumed to be linear isotropic elastic tissues. Menisci were assumed to be orthotropic elastic tissues (E_θ_ = 125*MPa*,E_R_ = E_Z_ = 27.5*MPa*,G_θ*R*_ = G_θ*Z*_ = 2*MPa*, G_RZ_ = 10.34,V_θ*R*_ = V_θ*Z*_ = 0.1*and*V_RZ_ = 0.33). Ligaments were defined as isotropic hyperelastic tissues using strain energy functions. The Veronda-Westmann function was used to define the properties of the ACL and PCL (α_ACL_ = 0.3 MPa, β_ACL_ = 12.20, α_PCL_ = 0.18 MPa and β_PCL_ = 17.35) while the Mooney-Rivlin material model was used to define the mechanical properties of the MCL and LCL. The coefficients of the LCL were assumed to be identical to those of the MCL (C1 = 30.1 MPa and C2 = −27.1 MPa). A pre-strain of 3% was placed on the ACL. A frictionless sliding interface was defined between the femoral cartilage and tibial cartilage and between the femoral cartilage and top surface of the menisci to permit sliding without penetrating the surfaces. A tie connection was defined between the ligaments and corresponding bone insertions. The model was validated against data from the same cadaveric knee tested in a robotic simulator, resulting in an accuracy of 0.2 mm for joint translation and 5 N for ACL force.

### Simulation of Conventional ACLR Using Circular Tunnel

To simulate conventional ACLR, the original ACL was removed, and the femoral and tibial tunnels (diameter 7.5 mm) were created through the center of ACL insertion sites. The angles between the femoral tunnel axis and the axial and sagittal planes were 45° and 25°, respectively, and the angles between the tibial tunnel axis and the axial and sagittal planes were 65° and 25°, respectively (Yao et al., 2014). A four-strand hamstring tendon graft was simulated as being cylindrical in shape with a diameter of 7.5 mm and stiffness of 776 N/mm (Hamner et al., 1999). Titanium endoscrews (Young’s modulus = 110 GPa, and Poisson’s ratio *v* = 0.35) (Hung et al., 2014) with a diameter of 7.5 mm and length of 25 mm were used to secure the graft. The endoscrews were tied to the ends of the graft by its bottom surface and tied to the tunnel wall by its outer surface. Frictionless sliding was defined between the outer surface of the graft and the tunnel wall to allow relative motion between them without penetrating the surfaces.

### Simulation of ACLR Using Different Shapes for the Tunnel Aperture

**FIGURE 2:**
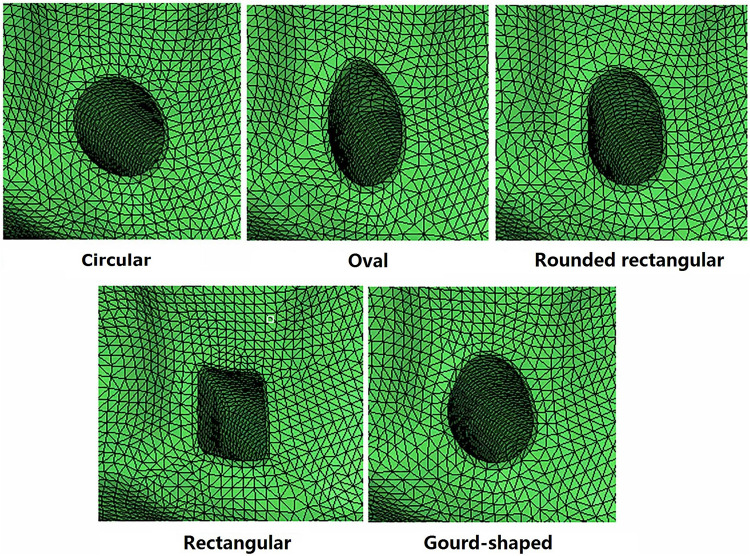
Different tibial tunnel shapes simulated for single bundle ACL reconstruction.

**FIGURE 3:**
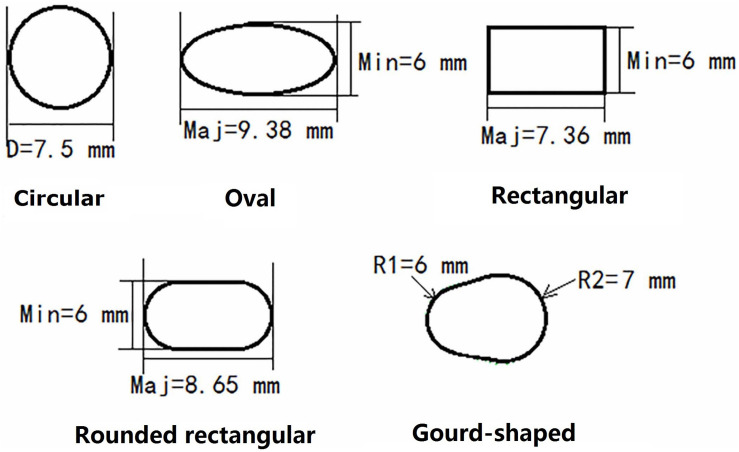
Dimensions of the various tunnel apertures created in the tibia.

For the models created with non-circular bone tunnel entrances, the femoral tunnel aperture was oval-shaped in all models because the majority of ACL femoral insertion sites have been reported as resembling this shape (Petersen and Zantop, 2006). However, the tibial tunnel aperture was modeled as being oval, rounded rectangular, rectangular or gourd-shaped, as shown in Figure 2. Similar to previous studies (Zhang et al., 2019), while changing the shape of the bone tunnel aperture, the cross sectional area of the main body of the tunnel was kept constant to ensure a close match with the diameter of the autogenous hamstring graft. In this study, the minor axes of each tunnel, regardless of shape, was set to be 6 mm, while the length of the major axes was calculated from the constant cross sectional area ($\pi \times {\left(\frac{7.5}{2}\right)}^{2})$ (Zhang et al., 2019). The dimensions of the various tibial tunnel apertures are shown in Figure 3. To perform the procedure, a cylindrical bone tunnel of diameter 7.5 mm was first created from the medial section of the tibia under the tibial plateau to a depth 10 mm distal from the tunnel aperture. The angle between the bone tunnel and the axial and sagittal planes was the same as that used in conventional ACLR. This tunnel was then adjusted and extended to form the different shapes of the aperture shown in Figure 2. The length and location of the endoscrew were kept the same as the conventional ACLR group.

### Simulation of ACLR Using Different Tunnel Diameters

Anterior cruciate ligament reconstruction using circular tunnels of diameter 7.5, 8.5, and 9.5 mm created in the tibia and femur was also simulated in this study to offer a wider scope of results for comparison against the results from the different tunnel shapes. The diameter of the grafts was correspondingly changed (7.5, 8.5, and 9.5 mm) to match the tunnel diameter.

### Loading Condition and Simulation Outputs

The loading conditions (Figure 1) were the same for all models: intact knee, conventional ACLR using a circular tunnel aperture, ACLR using other shapes for the tunnel aperture and ACLR using different tunnel diameters. The maximum anterior tibial load (103 N, 15% body weight), internal tibial moment (7.5 Nm, 1.1% body weight) and valgus tibial moment (6.9 Nm, 1% body weight) during normal walking (Kutzner et al., 2010) were applied to the models (top surface of the femur was fixed and the loadings were applied to the tibia shaft) at a joint flexion angle of 20°. This loading condition represents a worst-case situation for the ACL to be tensioned during walking. The following parameters were recorded from each model for comparison: joint kinematics including ATT and internal and valgus tibial rotation (ITR and VTR), ACL/graft force, and the maximum SED on the medial and lateral tibial cartilage.

## Results

### Joint Kinematics and ACL/Graft Force

As shown in Table 1, in comparison to the intact joint, conventional ACLR using a circular tunnel aperture resulted in an increase in ATT, ITR, and VTR, but the graft force was lower than the native ACL.

**TABLE 1:** Joint kinematics and ACL force at different joint states under a combined loading of 103 N anterior tibial load, 7.5 Nm internal tibial moment and 6.9 Nm valgus tibial moment at a joint flexion angle of 20°.

		**Anterior**	**Internal**	**Valgus**	**ACL/**
		**tibial**	**tibial**	**tibial**	**graft**
		**translation**	**rotation**	**rotation**	**force**
		**(mm)**	**(°)**	**(°)**	**(N)**
Intact joint		2.5	13	1	126
Conventional ACLR (D7.5)		3.0	14	2	106
Anatomical	Oval	3.1	14	2	103
single tunnel	Rounded rectangular	3.4	15	2	101
ACLR in	Rectangular	3.1	14	2	104
different tibial	Gourd-shaped	3.4	14	2	99
tunnel shapes					
Conventional ACLR (D8.5)		2.7	13	2	111
Conventional ACLR (D9.5)		2.4	13	1	117

*D7.5, D8.5, D9.5 represent conventional ACLR using a circular tunnel of diameter 7.5 mm, 8.5 mm and 9.5 mm, respectively.*

Changing the shape of the tunnel aperture resulted in a lower graft force than the circular tunnel but caused an increase in ATT, while ITR and VTR showed little change. The rounded rectangular and gourd-shaped tibial apertures produced the greatest ATT, while the rounded rectangular shape also produced the greatest ITR. In terms of the force in the graft, the gourd-shaped tibial tunnel had the lowest value, which was followed by the rounded rectangular shape. The force in the graft was greatest when using rectangular and oval tunnels, but the magnitude was still lower than the conventional ACLR group.

Increasing the diameter of the tunnel led to a decrease in joint kinematics and increase in graft force, bringing the values from the model with a 9.5 mm tunnel within range of the intact knee (Table 1). Similarly, using a tunnel diameter of 9.5 mm also restored the graft force to within 9 N of the intact ACL force (117 vs. 126 N).

### Strain Energy Density on the Articular Cartilage

**FIGURE 4:**
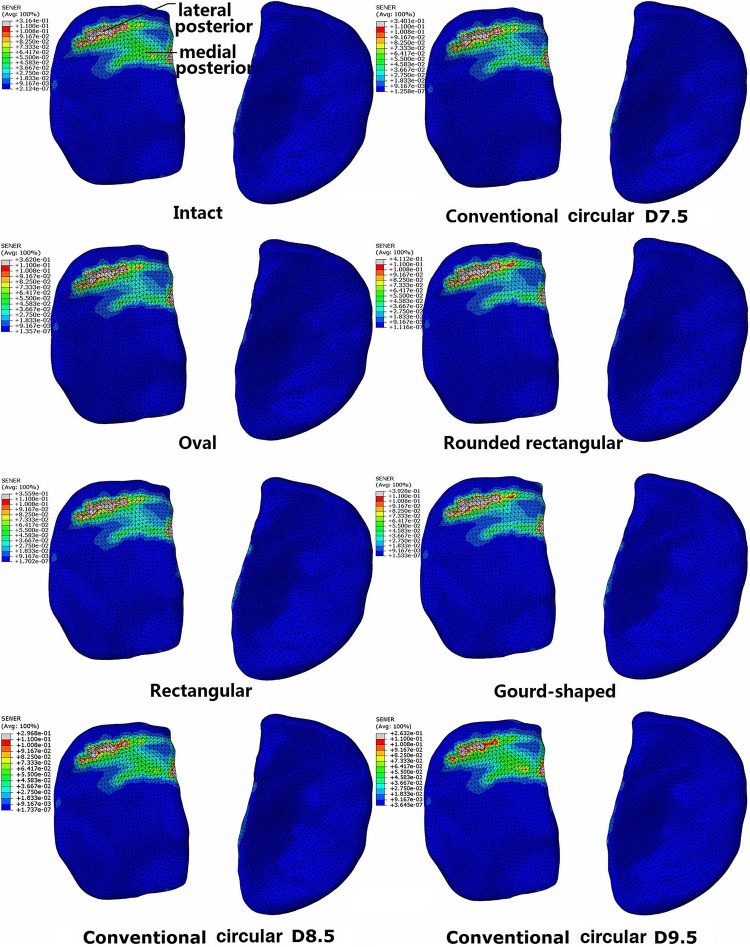
Distribution of strain energy density on the tibial cartilage at different joint states under a combined loading of 103 N anterior tibial load, 7.5 Nm internal tibial moment and 6.9 Nm valgus tibial moment at a joint flexion angle of 20°. The descriptions under the images (oval, rounded rectangular, rectangular, and gourd-shaped) represent the different shapes of the tibial tunnel apertures in the ACLR models. D7.5, D8.5, D9.5 represent conventional ACLR using a circular tunnel of diameter 7.5, 8.5, and 9.5 mm, respectively.

The distribution of SED on the tibial cartilage for each model is displayed in Figure 4. The intact joint had a concentrated SED at the posterior section of the lateral tibial cartilage. Regardless of the tunnel shape, all ACLR models showed higher SED at the lateral posterior section (see Figure 4) and lower SED at the medial posterior section. The rounded rectangular and gourd-shaped aperture groups had the most drastic change in SED. Increasing the tunnel diameter to 8.5 and 9.5 mm lowered the SED at the lateral posterior section of the lateral tibial cartilage. Also, compared with the lateral tibial cartilage, the SED on the medial cartilage was much lower.

The maximum SED on the tibial cartilage is recorded in Table 2. In comparison to the intact knee, conventional ACLR using a circular tunnel led to a decrease in the maximum SED on the medial tibial cartilage but an increase on the lateral side. Changing the shape of the tunnel magnified this change, with the highest maximum SED occurring on the lateral tibial cartilage when using a rounded rectangular tunnel, followed by a gourd-shaped tibial tunnel. Among all the different shapes for the tunnel aperture, the rectangular tunnel had the closest SED to the intact joint. In contrast, using a larger tunnel diameter noticeably decreased the maximum SED on the lateral tibial cartilage. In comparison to ACLR with a 7.5 mm tunnel, using a tunnel diameter of 8.5 and 9.5 mm resulted in a lower articular SED, and the results were even slightly lower than the intact joint.

**TABLE 2:** The maximum strain energy density (SED) on the medial and lateral tibial cartilage at different joint states under a combined loading of 103 N anterior tibial load, 7.5 Nm internal tibial moment and 6.9 Nm valgus tibial moment at a joint flexion angle of 20°.

		**Maximum SED on**	**Maximum SED on**
		**the medial articular**	**the lateral articular**
		**surface (10^3^J/m^3^)**	**surface (10^3^J/m^3^)**
Intact joint		38	316
Conventional ACLR (D7.5)		33	340
Anatomical	Oval	19	362
single tunnel	Rounded rectangular	24	411
ACLR in	Rectangular	32	356
different tibial	Gourd-shaped	35	393
tunnel shapes			
Conventional ACLR (D8.5)		28	297
Conventional ACLR (D9.5)		29	263

*D7.5, D8.5, D9.5 represent conventional ACLR using a circular tunnel of diameter 7.5 mm, 8.5 mm and 9.5 mm, respectively.*

## Discussion

The key finding of this study was that changing the shape of the tibial bone tunnel aperture in ACLR from a traditional circular shape to any of the shapes presented in Figure 2 did not better restore the joint kinematics, graft force or SED on the tibial cartilage. However, increasing the tunnel diameter was shown to produce joint biomechanics closer to the intact joint, which was consistent with previous studies (Westermann et al., 2013).

Compared to the intact joint, the results for ATT, ITR, and VTR increased after conventional ACLR, while the force in the graft was lower than the native ACL. These results were in agreement with those reported by Yagi et al. (2002), and also supported the proposal that conventional ACLR using a circular tunnel aperture could not sufficiently restore joint stability to bring it within range of the intact knee. Also, the altered loading environment could be expected to induce long-term OA in the knee joint. The lower force in the graft indicated increased load bearing by other tissues in the knee, which could put these tissues at risk of secondary injury.

Increasing the tunnel diameter also resulted in improved joint stability and lower stress on the articular surface, which was consistent with previous literature (Westermann et al., 2013). These results implied that a larger tunnel diameter might be beneficial for delaying the occurrence of long-term OA following ACLR.

Changing the shape of the tunnel aperture from circular to an alternative shape shown in Figure 2 reduced the joint stability and resulted in lower graft force, and also imposed greater stress on the lateral tibial cartilage. Among the tunnel shapes simulated in this study, the gourd shape and rounded rectangular shape produced the greatest ATT and the lowest graft force, which are not desirable traits for treating a ruptured ACL.

Compared with the intact knee, the use of a 7.5 mm circular tunnel led to a posterior shift in the articular SED concentrations (Figure 4). Along with the altered joint kinematics, the increased SED at the lateral posterior section could be a result of the greater ATT and ITR. This may explain why the rounded rectangular tunnel shape produced the greatest ITR and maximum articular SED simultaneously. Changing the tunnel aperture from circular to an alternative non-circular shape resulted in a considerable increase in maximum SED on the lateral articular cartilage (Table 2), indicating that these alternative shapes might induce an earlier onset of OA.

Using one of the alternative non-circular tunnel shapes did not improve joint function. It is conjectured by the authors that the increased SED may be due to the constant cross-sectional area of the tunnel in this study. When changing the cross sectional shape, the span of the insertion site is elongated in one direction, but the perpendicular length is shortened. However, increasing the diameter of the bone tunnels was shown to offer better joint stability and reduce stress on the articular surface. Thus, it can be concluded that increasing the area of the bone insertion sites of the ACL is more effective for improving the outcome of ACLR than changing the shape of the tunnel aperture. However, in such situations with a large tunnel, an artificial graft would likely be required since the diameter of autografts cannot generally be tailored.

There are some limitations to this study to be noted. (i) The cadaveric sample used in this study had a rounded triangular shape for the tibial insertion site of the ACL, which according to previous studies is representative of approximately half of all tibial insertions. However, this also means the other half of the population is not adequately represented in this study. (ii) To control the study parameters and ensure the tunnel shape is the only variable, the stiffness and fixation method of the grafts were the same for all simulation groups. An endoscrew was used to securely fix the graft into the bone tunnel and it was assumed that no relative motion occurred between the screw and the tunnel wall, as well as between the screw and the graft. This would theoretically result in a more stable joint than is actually experienced clinically. (iii) The cross-sectional area of the tunnel was maintained constant while changing the tunnel shape.

In conclusion, increasing the diameter of a traditional circular tunnel may be more effective for restoring joint biomechanics after ACLR than changing the shape of the tunnel aperture.

## Data Availability Statement

All datasets generated for this study are included in the article/supplementary material.

## Author Contributions

HW and C-KC conceptualized the study. HW and MZ conducted the model calculation. All authors participated in data analysis and writing and editing the manuscript.

## Conflict of Interest

The authors declare that the research was conducted in the absence of any commercial or financial relationships that could be construed as a potential conflict of interest.

## Abbreviations

ACL: anterior cruciate ligament
ACLR: anterior cruciate ligament reconstruction
ATT: anterior tibial translation
ITR: internal tibial rotation
LCL: lateral collateral ligament
MCL: medial collateral ligament
OA: osteoarthritis
PCL: posterior cruciate ligament
SED: strain energy density
VTR: valgus tibial rotation
